# Personalized medicine with IgGAM compared with standard of care for treatment of peritonitis after infectious source control (the PEPPER trial): study protocol for a randomized controlled trial

**DOI:** 10.1186/s13063-019-3244-4

**Published:** 2019-03-04

**Authors:** Christina Kalvelage, Kai Zacharowski, Artur Bauhofer, Ulrich Gockel, Michael Adamzik, Axel Nierhaus, Peter Kujath, Christian Eckmann, Mathias W. Pletz, Hendrik Bracht, Tim-Philipp Simon, Michael Winkler, Detlef Kindgen-Milles, Markus Albertsmeier, Markus Weigand, Björn Ellger, Maximilian Ragaller, Roman Ullrich, Gernot Marx

**Affiliations:** 1 Center for Translational and Clinical Research Aachen, RWTH Aachen University, Pauwelsstr. 30, 52074 Aachen, Germany; 2 Department of Anaesthesiology, Intensive Care Medicine and Pain Therapy, University Hospital Frankfurt, Frankfurt, Germany; 3 Corporate Medical Affairs, Biotest AG, Landsteinerstr. 5, 63303 Dreieich, Germany; 4 Medical Affairs Central Europe, Biotest AG, Landsteinerstr. 5, 63303 Dreieich, Germany; 5 Department of Anesthesiology, Intensive Care Medicine and Pain Therapy, Ruhr-Universität Bochum, In der Schornau 23–25, 44892 Bochum, Germany; 6 Department of Intensive Care Medicine, University Hospital Hamburg-Eppendorf, Martinistraße 52, 20246 Hamburg, Germany; 7 Department of Surgery, University of Schleswig-Holstein, Ratzeburger Allee 160, 23538 Lübeck, Germany; 8 Department of General, Visceral, and Thoracic Surgery, Klinikum Peine Academic Hospital of Medical University Hannover, Virchowstraße 8h, 31226 Peine, Germany; 9 Center for Infectious Diseases and Infection Control, Jena University Hospital, Erlanger Allee 101, 07747 Jena, Germany; 10 Department of Anaesthesiology, University Ulm, Albert Einstein Allee 23, 89081 Ulm, Germany; 11 Department of Intensive Care and Intermediate Care, University Hospital RWTH Aachen, Pauwelsstr.30, 52074 Aachen, Germany; 12 Department of General, Visceral and Transplantation Surgery, Hannover Medical School, Carl-Neuberg-Str. 1, 30625 Hannover, Germany; 13 Department of Anaesthesiology, Düsseldorf University Hospital, Moorenstr. 5, 40225 Düsseldorf, Germany; 14 Department of Anaesthesiology, Ludwig-Maximilians-University of Munich, Marchioninistrasse 15, 81377 Munich, Germany; 15 Department of Anesthesiology, Heidelberg University Hospital, Im Neuenheimer Feld 110, 69120 Heidelberg, Germany; 16 Department of Anesthesiology, Intensive Care and Pain Medicine, Klinikum Westfalen, Am Knappschaftskrankenhaus 1, 44309 Dortmund, Germany; 17 Department of Anesthesiology and Intensive Care Medicine, Technical University Dresden, Fetscherstraße 74, 01307 Dresden, Germany; 18 Department of Anaesthesia, Critical Care and Pain Medicince, Medical University of Vienna, Währinger Gürtel 18-20 / 9i, 1090 Vienna, Austria

**Keywords:** Personalized medicine, Biomarkers, Pentaglobin, IgGAM, Sepsis, Peritonitis, Severe bacterial infection

## Abstract

**Background:**

Peritonitis is responsible for thousands of deaths annually in Germany alone. Even source control (SC) and antibiotic treatment often fail to prevent severe sepsis or septic shock, and this situation has hardly improved in the past two decades. Most experimental immunomodulatory therapeutics for sepsis have been aimed at blocking or dampening a specific pro-inflammatory immunological mediator. However, the patient collective is large and heterogeneous. There are therefore grounds for investigating the possibility of developing personalized therapies by classifying patients into groups according to biomarkers. This study aims to combine an assessment of the efficacy of treatment with a preparation of human immunoglobulins G, A, and M (IgGAM) with individual status of various biomarkers (immunoglobulin level, procalcitonin, interleukin 6, antigen D-related human leucocyte antigen (HLA-DR), transcription factor NF-κB1, adrenomedullin, and pathogen spectrum).

**Methods/design:**

A total of 200 patients with sepsis or septic shock will receive standard-of-care treatment (SoC). Of these, 133 patients (selected by 1:2 randomization) will in addition receive infusions of IgGAM for 5 days. All patients will be followed for approximately 90 days and assessed by the multiple-organ failure (MOF) score, by the EQ QLQ 5D quality-of-life scale, and by measurement of vital signs, biomarkers (as above), and survival.

**Discussion:**

This study is intended to provide further information on the efficacy and safety of treatment with IgGAM and to offer the possibility of correlating these with the biomarkers to be studied. Specifically, it will test (at a descriptive level) the hypothesis that patients receiving IgGAM who have higher inflammation status (IL-6) and poorer immune status (low HLA-DR, low immunoglobulin levels) have a better outcome than patients who do not receive IgGAM. It is expected to provide information that will help to close the knowledge gap concerning the association between the effect of IgGAM and the presence of various biomarkers, thus possibly opening the way to a personalized medicine.

**Trial registration:**

EudraCT, 2016–001788-34; ClinicalTrials.gov, NCT03334006. Registered on 17 Nov 2017.

**Trial sponsor:** RWTH Aachen University, represented by the Center for Translational & Clinical Research Aachen (contact Dr. S. Isfort).

**Electronic supplementary material:**

The online version of this article (10.1186/s13063-019-3244-4) contains supplementary material, which is available to authorized users.

## Background

### Peritonitis

In Germany, around 10,000 patients are treated annually for peritonitis [1]. Owing to the copious blood supply of the peritoneum, an infection can rapidly spread to the entire organism, causing potentially life-threatening damage. Peritonitis is classified as follows [2]: primary (hematogenic in children, bacterial in adults, or following tuberculosis or gonorrhea); secondary (post-operative, post-traumatic, or due to perforation); tertiary (owing to immune deficiency, usually avirulent); quaternary (due to intra-abdominal abscess, or hospital-acquired or catheter-associated infection); and specific forms due, e.g., to *Candida* infection or chemical effects.

Intra-abdominal infections are treated by standard methods of surgery and intensive care. As peritonitis can rapidly lead to bacteremia and sepsis, infectious source control and antibiotic treatment remain the methods of choice to limit the spread of bacteria and the secretion of their toxins. Even after source control and adequate antibiotic treatment, severe sepsis or septic shock can occur, so that the lethality associated with peritonitis is still high and, despite all medical progress, has hardly been reduced in the past 20 years. With an overall mortality rate between 30% and 40% depending upon the population observed, (post-operative) secondary peritonitis remains one of the deadliest diseases in the intensive care unit (ICU) [3].

Hitherto, most studies of immunomodulatory therapies for sepsis have aimed at blocking, or dampening, a specific pro-inflammatory immunological mediator within a large and heterogeneous patient collective. These—in some cases large-scale—studies generally gave negative results. The failure of “single-mediator” strategies appears to have lain in the pleiotropy and redundancy of corresponding mediator/cytokine networks and in inadequate immunological characterization of the study patients under treatment. Adequate immunological characterization of study patients is necessary, as the justification of immunological interventions demands evidence of immunological efficiency. This is especially the case for the highly heterogeneous collective of sepsis patients, and it lies behind the urgency of identifying biomarkers that allow immunological characterization and prognostic assessment.

In the future, a “personalized” therapy may be made possible by classifying patients into groups according to biomarkers (currently, about 30 medicinal preparations marketed in Germany require such pre-diagnosis and another ten recommend it). The use of such tests may allow better prediction of whether—and in some cases how—a patient will respond to a given treatment. A further important consideration is maintaining the balance between pro- and anti-inflammatory immune actions, avoiding over-response on the one hand and immune exhaustion on the other [4].

In sepsis, biomarkers are used for diagnosis, risk stratification, progress-monitoring, and adaptation of therapy. Apart from unspecific inflammation and acute-phase markers (leukocyte count; C-reactive protein (CRP)), biomarkers with high specificity and sensitivity are available: procalcitonin (PCT) and antigen D-related human leukocyte antigen (HLA-DR) [5]. The frequently used CRP is only of limited value in sepsis on account of its long induction time (up to 48 h) and the persistence of elevated levels, both in early diagnosis and for assessment of the course and prognosis of disease [6, 7]. These conventional markers allow only restricted inferences to be drawn about the immunological situation in sepsis cases.

The following biomarkers have been identified as being able to aid decision-making:

- PCT: With its short induction time of 1–2 h and up to 10^4^-fold increase [8–10], serum PCT possesses high sensitivity (89–96%) and specificity (78–94%) [7]. It can be used to steer antibiotic treatment [11], which in a study shortened from 12.9 to 5.8 days [12, 13].
- Interleukin 6 (IL-6): A pro-inflammatory cytokine expressed by monocytes/macrophages, endothelial cells, keratinocytes, and fibroblasts. It has a rise time of only ~ 2 h [14] and is correlated with the severity of sepsis [15, 16].
- HLA-DR: The standardized measurement of HLA-DR expression indicates the level of monocyte functions and allows immunological characterization of the patient. Patients identified as having restricted immunological function can profit from an immunomodulatory therapeutic strategy [17, 18].
- Immunoglobulins (IgG, IgM, IgA): Recent studies have revealed clear correlations between survival probability and levels of IgG, IgM, und IgA (singly or in combination) in severe sepsis and septic shock [19, 20]. Patients with depressed Ig levels have a poorer chance of survival. One study revealed a particularly strong survival advantage (factor 5.2) when IgG1, IgA, and IgM exceeded the following values: IgG1 > 300 mg/dl, IgM > 35 mg/dl, IgA > 150 mg/dl [20]). This agrees with other recent study results on the kinetics of IgM concentration during the course of sepsis [21].
- Adrenomedullin (ADM): Adrenomedullin has a vasodilatatory effect and influences several systems (circulation, heart, hormone secretion, and respiration [22–24]). It plays a major part in initiating the hyperdynamic response in early-phase sepsis, and its level is correlated with the severity of disease. The available data suggest that the level of biologically active ADM may be usable as a biomarker for septic shock [25]; further studies are called for to assess its value.
- NF-κB1: The transcription factor NF-κB1 mediates the cell’s acute response to pathogenic and inflammatory stimuli, activating many of the so-called “immediate early genes” (tissue factors, endothelins, cytokines) involved in sepsis, and regulating some 500 different genes in all along with the processes of immune response, cell proliferation, and apoptosis. The activation of NF-κB1 is believed to be critical for the initiation of inflammation. One possible reason for the failure of sepsis studies may be traceable to the genetic polymorphism of NF-kB1 [26–28]. Analysis of NF-κB1 polymorphism is the object of current research.
- Identification of pathogens from blood and abdominal secretions (pathogen spectrum). A standardized analysis of the microbiological pattern from ascitis and from blood will be performed with the Curetis Unyvero molecular biology system (Curetis GmbH; Holzgerlingen, Germany). Treatment of septic patients is dependent on the type of microorganism(s) [29] and of resistance(s) [30]. The effect of IgGAM on different microorganisms in septic patients will be explored.

For a long time, abdominal sepsis was thought to be purely hyperinflammatory in nature. It is now known that every hyperinflammation induces a compensatory hypoinflammation [31]. These do not alternate; rather, hypo- and hyperinflammation can overlap in time and sepsis is to be regarded as a dysregulation of inflammation.

### Study treatment

The efficacy of treatment with IgGAM (Pentaglobin®) was demonstrated in a double-blind study of 56 patients with peritonitis accompanied by severe sepsis or septic shock [32]. A meta-analysis of 560 patients in eight studies revealed a pooled relative risk of mortality of 0.64, and the pooled effect of seven studies on 932 patients was 0.85 [33]. More recent studies have shown that a depressed IgM level in sepsis (including peritonitis) is associated with poorer outcomes [34]. In severe pneumonia, the CIGMA study showed that IgM substitution reduced the mortality in the subgroup of patients with low IgM or high CRP ([35]).

Despite this, the available information is inadequate [36], so that expert recommendation (on the basis of the meta-analysis [33]) corresponded only to level C in the German sepsis guideline. For peritonitis no recent data are available, and the present study is intended to fill this gap. It is also intended to investigate the possible relevance of various biomarkers (see above) in identifying subgroups of patients who could especially profit from treatment with IgGAM. This could provide the basis for a large-scale (phase III) study. The biomarker may also help in designing personalised adjuvant treatments with IgGAM in the indication of peritonitis.

### Risks and benefits

IgGAM has an immunoregulatory effect, i.e., hyper- und hypoinflammation are both enhanced. Pathogens are eliminated and endotoxins are neutralised. Studies [32, 37] have shown that the administration of IgGAM as an adjuvant treatment can improve the survival of peritonitis patients.

Immunoglobulins are natural, human proteins, the clinical use of which has been well established since the 1980s [38].We consider the high dose level to be justified on account of the extremely poor condition of these patients (Sequential Organ Failure Assessment (SOFA) score ≥ 8 [39]); the lower levels given in the Specification of Product Characteristics [40] are “recommendations” that may “serve as a guide”. In earlier studies [32, 35] the higher dose levels to be used in the present study were also successfully applied.

The risk of viral infection is considered to be negligible and not to warrant testing beyond the manufacturer’s standard procedures. The only invasive study tests involve drawing a total of 50 ml blood over 5 days. Thus, the overall risk to the patients is very small and is favourable in the light of the expected possible benefits to the study patients and the gain in knowledge to be acquired.

### Objectives and assessment criteria

The purpose of this study is to evaluate the adjuvant IgGAM treatment in respect of:

- An improvement of the outcome for the patient’s peritonitis. This will be investigated by using scores such as the multiple organ failure (MOF) score [41] (as modified [42], Additional file 1) and Sequential Organ Failure Assessment (SOFA) score as well as the Mannheim Peritonitis Index (MPI; a validated index used commonly in Germany [43] and in several other European countries [44]) and survival data.
- Identification of biomarkers (Ig level, PCT, IL-6, HLA-DR, NF-κB1, ADM, pathogen spectrum) to identify patient subpopulations that profit most from treatment with IgGAM. Such patients will comprise the basis for a further study, which will be a randomized, controlled, double-blind trial to demonstrate the value of this treatment.
- Furthermore, these biomarkers are expected to help with developing a “personalized” adjuvant therapy with IgGAM in the indication of peritonitis.

#### Primary endpoint

Improvement of the mean MOF score on day 7 expressed as the difference in MOF score between day 7 and day 0 (Δ [MOF Day 7 − Day 0]). The analysis will be adjusted for the baseline MOF score (day 0). The primary analysis will be performed with the PP population (see definition below).

The MOF score is determined in the morning, with the following scoring for each organ (lungs, heart, kidneys, liver, blood): normal function, 0; dysfunction, 1; individual organ failure, 2. An aggregate score greater than 4 implies multi-organ failure. Patients who die will be assigned the maximum score of 10 and will be included in the population assessment.

#### Secondary endpoints

- Overall 28-day survival
- Overall 90-day survival
- Improvement in MOF score on day 5
- Relative number of patients with multi-organ failure (i.e., > 4 MOF points) on day 7.

#### Additional study variables

- Time course of the biomarkers (PCT, IL-6, HLA-DR, ADM, IgM, IgG, IgA), the SOFA score, the MPI, the surrogate variables for organ dysfunction and survival according to Heyland et al. [45], and vital signs.
- Influence of the biomarkers NF-κB1, ADM, and pathogen spectrum upon the improvement of the mean MOF score on day 7.
- Comparison of the MOF score with other scores, such as the SOFA score, for assessment of organ dysfunction.

## Methods/design

### Study design

This multicentric, prospective clinical study will be conducted according to a controlled, randomized, two-arm, parallel-group design. No blinding will be performed. The study is planned to be conducted in 14 centers in Germany and Austria.

### Study patients

The following eligibility criteria were selected to include patients of either sex, with abdominal surgery developing peritonitis, with severe sepsis (SOFA score), IL-6 (sustained inflammation), and with concurrent guideline-appropriate therapy (timelines of source control, IgGAM treatment, and antibiosis). We decided to use the SOFA score as an inclusion criterion to assure a homogenous patient population with a certain level of disease severity. Additionally, the SOFA score is used worldwide, being included in the current Sepsis Guideline; this makes it more common today on ICUs [46], which in turn simplifies the identification of potential study patients. The MOF score is less commonly applied, but it focuses on the failure of different organ systems. Besides scoring, the MOF score also allows identification of the organ system that benefits most from the IgGAM treatment and identification of multi-organ failure with an aggregated score greater than 4, which makes it very well suited to serve as a primary outcome criterion [42]. The eligibility criteria include only the IL-6 and not the immunoglobulin levels, since short-term immunoglobulin determination cannot be implemented in practice as it is not available 24 h a day at all study sites.

#### Inclusion criteria

1. Peritonitis.
2. Time of source control intervention: within 6 h of determination of the indication (defined as the time of placing the patient on the list for operation or minimally invasive measure).
3. Sepsis and septic shock (according to the current sepsis guideline [47]).
4. SOFA score of 8 or above.
5. IL-6 level of 1000 pg/ml or above.
6. Antibiotic treatment begun within 12 h after entry to the ICU.
7. Written informed consent by the patient or if appropriate by his/her legal representative or the consulting physician.

#### Exclusion criteria

1. Life expectancy less than 90 days because of medical circumstances that are not related to the patient’s peritonitis or sepsis/septic shock.
2. Pregnancy, breast-feeding.
3. Age below 18 years.
4. Known chronic kidney insufficiency requiring dialysis (creatinine ≥ 3.4 mg/dl or creatinine clearance ≤ 30 mL/min/1.73 m^2^).
5. Pancreatitis or mediastinitis.
6. Body mass index > 40 kg/m^2^.
7. Presence of any counter-indication against the study medication.
8. Participation in any other mediation study within the previous 30 days.
9. A dependency on or a professional relationship with the sponsor or the investigator.
10. Commitment of the patient to any resident institution by order of any court or authority.

### Interventions

The control group will receive standard-of-care treatment, i.e., the IgGAM is an add-on treatment in this study. The use of placebo instead of standard therapy would further minimize possible bias potential and increase the quality of the study accordingly. Nevertheless, for reasons of feasibility, we have initially opted for an open design comparing IgGAM against standard care, especially since the present study was primarily designed to serve as a solid basis for a larger blinded follow-up study; in such a study, placebo controls would be used to investigate explicitly personalized medicine with IgGAM and to show an improvement in mortality with IgGAM treatment. The active study treatment is IgGAM, a preparation containing human immunoglobulins G, A, and M. The preparation to be provided contains 50 mg/ml human plasma proteins, of which ≥ 95% are immunoglobulins: IgM 6 mg, IgA 6 mg, and IgG 38 mg. The IgG subclass distribution is IgG1 ~ 63%, IgG2 ~ 26%, IgG3 ~ 4%, IgG4 ~ 7%. IgGAM is administered by continuous infusion over 5 days at a dose level of 0.4 ml/kg body weight per hour, until a total dose of 7 ml/kg on that day has been reached; administration will then be stopped and recommenced on the following day, until administration has been completed for 5 consecutive days.

### Course of the study

**Fig. 1:**
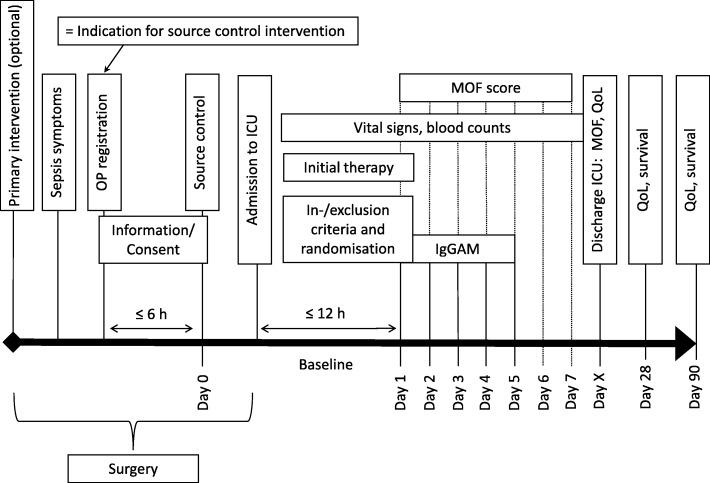
Course of the study. *ICU* intensive care unit, *IgGAM* preparation containing immunoglobulins G, A, and M, *MOF* multiple organ failure, *OP* operation , *QoL* quality of life

**Fig. 2:**
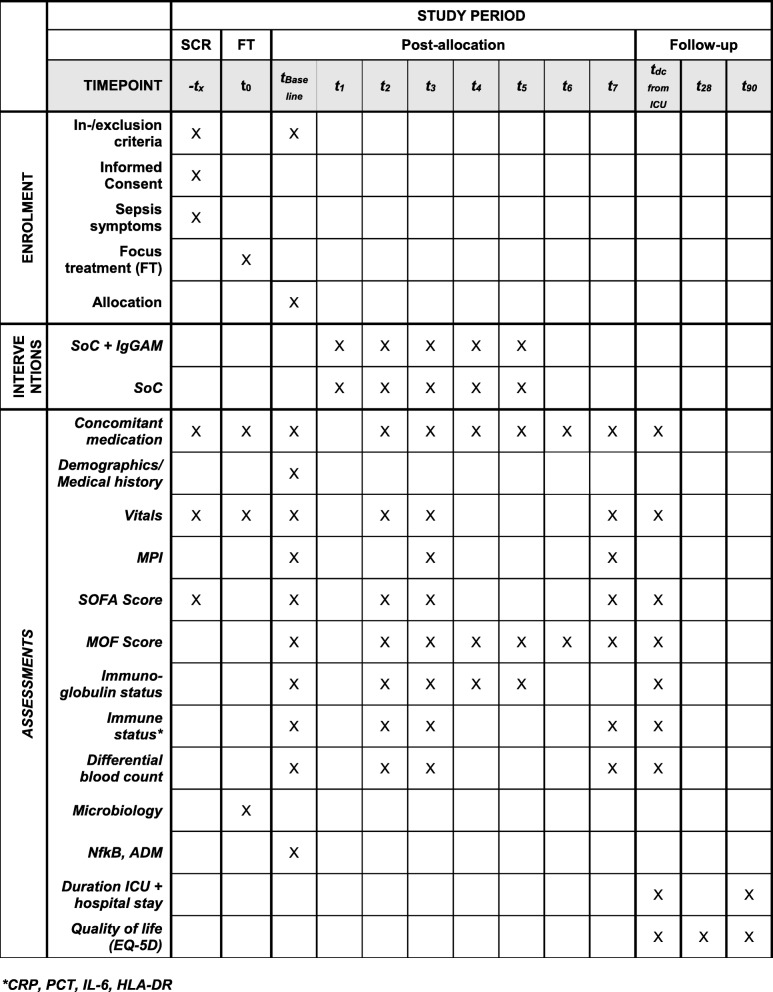
SPIRIT figure. For abbreviations see text

The study procedures are summarized in Figs. 1 and 2.

As soon as interventional source control for peritonitis-induced sepsis is indicated, screening (SCR) will commence. Patients will be informed about the study and written informed consent to participate will be requested by the investigator. If a patient is unable to give consent, he may be represented by his legal representative, such as a court-appointed carer or an authorized proxy. If no legal representative or court-appointed caregiver is available, then a study-independent consultant physician can provide consent on the patient’s behalf, though only if a review of the overall circumstances reveals clear positive evidence that the presumed will of the patient is in favor of participating in the study. As soon as the patient is able to consent, the examiner will inform him about the study, ask for his written consent and thus give him the opportunity to withdraw consent.

Empirical antibiotic treatment will be started with carbapenem or piperacillin/tazobactam; any other antibiotic must be documented along with the reason for choosing it. Interventional source control must begin within the 6 h after registration for the operation: successful control must be documented. Within the first hour after source control, guideline-compliant initial therapy is to be conducted in the ICU: acquisition of material for microbiological diagnosis, placing of a central vein catheter, invasive blood-pressure measurement, volume therapy, and empirical antibiotic treatment as above. Baseline is defined as the time point immediately after transfer of the patient to the ICU after interventional source control (before the first administration of IgGAM). At this point the inclusion and exclusion criteria will be checked and the patient, if eligible to participate, will be randomly allocated to the verum arm (133 patients, standard-of-care treatment plus IgGAM) or the control arm (67 patients, standard-of-care treatment only). A computer-generated block randomization list, stratified by center, with a control:intervention ratio of 1:2 is used. Block size is not revealed to the centers. Unbiased randomization is ensured by using a centralized online system provided and maintained by the sponsor.

For patients in the verum arm, IgGAM will be administered as described above, starting within 12 h of entry to the ICU; the day of this first administration is defined as day 1 (for patients in the control arm, day 1 is the day when they would have received IgGAM). IgGAM will be administered on days 1–5. The treatment phase ends on day 7; follow-up examinations are to be conducted on the day of discharge from the ICU and on days 28 and 90.

For all patients, the following information will be recorded: demography and anamnesis (baseline); concomitant medication up to discharge from the ICU; MPI (baseline, days 3 and 7); SOFA score (screening, baseline, days 2, 3, and 7, and at discharge); MOF score (baseline, days 2–7, and at discharge); vital signs (screening, source control on day 0, baseline, days 2, 3, and 7, and at discharge); adverse events (baseline to day 7 and at follow-up); quality of life (using the EQ QLQ 5D questionnaire; at discharge and on days 28 and 90); duration of stay in hospital and in the ICU.

Samples will be taken on days 2–5 and 28 for assessment of the following parameters: microbiological markers from ascites cells, including pH measurement of ascites, and from blood culture (day 0); NF-κB1 and ADM (baseline); CRP, PCT, IL-6, HLA-DR, differential blood count (baseline, days 2, 3, and 7, and at discharge); Ig status (baseline, days 2–5, and at discharge).

### Independent Adjudication Committee

For all patients, the adequacy and the course of peritonitis treatment (antibiotic treatment and source control) will be reviewed by an independent committee, the members of which are not otherwise involved in the study and will include experts in intensive care, surgery, and microbiology.

### Statistical analysis

#### Analysis sets

The intent-to-treat (ITT) population will comprise all patients included in the study. This will also be the population used for safety analysis. The primary analysis of efficacy will be conducted with a subset of the ITT population comprising only those patients whose treatment was considered by the independent Adjudication Committee (see above) to have been adequate; this subset we term the per protocol (PP) population.

#### Primary analysis

The study will investigate the hypothesis that patients receiving IgGAM who have higher inflammation status (IL-6 ≥ 1000 pg/ml, CRP ≥ 70 mg/L) and poorer immune status (HLA-DR expression ≤ 8000 molecules per monocyte; IgG1 < 300 mg/dl, IgM < 35 mg/dl, IgA < 150 mg/dl) have a better outcome than patients who do not receive IgGAM. This hypothesis is based on various clinical studies: Bermejo-Martin et al. [20] demonstrated that the combined presence of low levels of the endogenous immunoglobulins IgG1, IgM, and IgA in plasma is associated with reduced survival in patients with severe sepsis or septic shock. In parallel, Welte et al. [35] supported improved outcome regarding mortality with immunoglobulins in subsets of sCAP patients with elevated CRP, reduced IgM, or both. Both studies indicated that the assessment of the concentrations of these immunoglobulins could improve the results of treatment with exogenous immunoglobulins in patients with sepsis.

The primary endpoint (improvement in MOF score on day 7) will be analyzed by comparing the two treatment arms using mixed linear models.

A greater improvement of the mean MOF score on day 7 in the IgGAM-treated group by at least 0.8 points is expected. Afterwards the same analysis will be performed with the overall ITT population. The dependent variable will be the change in MOF compared with baseline (in the primary comparison: between baseline and day 7). Fixed independent variables will be treatment group and visit; baseline MOF will be a covariate and study center a random effect. Interaction between treatment and visit will be investigated. Restricted maximum-likelihood estimation and autoregressive covariance structure will be used. Sensitivity analyses will also be performed.

#### Secondary analyses

All other analyses will be at the descriptive level; results will be tabulated and nominal *p* values calculated for the ITT and PP populations. Relative survival will be analyzed by the Kaplan–Meier method. Cox regression models will be used, with treatment group and baseline MOF as independent variables. The numbers of patients with multi-organ failure will be analyzed by Poisson regression. Subgroup analyses are planned, using the subgroups ‘normal’ and ‘low’ IgM (cut off < 0.8 g/dl) and CRP (cut-off ≥ 70 mg/dl).

### Sample size

The following assumptions are made: significance level α = 0.05; power = 90%; randomization ratio verum:control 2:1; in MOF score 3.0 (verum) vs 3.8 points (control), each with a standard deviation of 1.5 points. A *t*-test model led to *N* = 114 in the active treatment group and 57 in the control group. To allow for drop-outs from the PP population, an additional margin of ~ 13% is allowed, giving *N* = 133 in the active-treatment group and 67 in the control group (total 200).

### Data management and quality assurance

All data will be collected in an electronic case report form, including edit checks, developed by a qualified data manager of the sponsor (RWTH Aachen University). The sponsor will furthermore ensure data validation, data verification, and compliance through regular monitoring visits according to ICH-GCP. The investigator will be required to authorize audits and inspections by competent surveillance authorities.

## Discussion

In recent decades there has been little progress in developing effective treatments for sepsis and septic shock in connection with secondary peritonitis. With a survival rate between 60% and 70% depending on the population observed, there is a clear unmet need for new approaches to this problem. Approaches to this have in recent years included the suppression of pro-inflammatory factors, including anti-TNF, anti-endotoxin, and anti-IL-6, and interference with the coagulation system, with, e.g., anti-thrombin III and activated protein C (drotrecogin alfa) [48–50] but neither could be shown to have clinically demonstrable efficacy.

In this study the PP population is used for the primary analysis, i.e., considering only patients for whom it is confirmed by the independent Adjudication Committee that they received treatment in respect of the mainstay of peritonitis treatment, i.e., successful source control and early adequate antibiotic treatment. These two measures are a prerequisite for any supportive treatment such as the application of IgGAM. The correct and timely application of IgGAM will be also assessed by the Adjudication Committee. A similar approach for primary analysis was used by Solomkin et al. [51], in a randomized controlled study of complicated abdominal infection, for assessment of non-inferiority of eravacycline compared with ertapenem. In that study, of the 541 patients randomized, only 465 patients received adequate treatment and could be included in the predefined primary analysis.

The sample size in the present study is considered adequate to allow demonstration of statistical superiority of IgGAM over standard-of-care in peritonitis patients by the criteria of MOF, but is not adequate for mortality or subgroup analyses. Nevertheless, it is important to identify subgroups of patients who benefit most from IgGAM, to allow individualized treatment of these patients, probably identified by biomarkers assessed in this trial. The sample size in the present study is probably too small for such an analysis; however, the study was primarily designed to serve as a solid base for a larger blinded follow-up study with placebo controls to investigate explicitly personalized medicine with IgGAM and to show an improvement in mortality with IgGAM treatment. Another approach, made possible by increasingly exact and detailed analytical measurements, could be the adaptation of treatment to the individual patient, i.e., “personalized” therapy. For this reason it will be necessary to discover which patients will profit best from immunomodulatory treatment, and of which kind. It is therefore hoped that the present study will provide more information toward such therapies, by allowing correlations to be established between the patients’ response to treatment with IgGAM and the presence or absence of one or more of a number of pre-defined biomarkers.

## Trial status

The trial protocol has received a positive vote from the responsible ethics committee (Ethik-Kommission der Medizinischen Fakultät des Universitätsklinikums der RWTH Aachen, EK 156/17), from the German federal authority for trials involving biomedical products (Paul-Ehrlich-Institut, Langen, PEI 3104/01) and from the Austrian federal authority for trials (BASG 10621880). The first patient was recruited in November 2017. This manuscript is written in accordance with the SPIRIT Statement (Additional file 2).

## Additional files

Additional file 1: MOF score as modified by Lefering et al. [42]. (PDF 101 kb)

Additional file 2: SPIRIT checklist (2013 version)*.* (DOCX 48 kb)

## Abbreviations

ADM: Adrenomedullin
CRP: C-reactive protein
HLA-DR: Human leucocyte antigen, antigen D-related
ICU: Intensive care unit
Ig: Immunoglobulin
IgGAM: Preparation containing immunoglobulins G, A, and M
IL: Interleukin
ITT: Intention-to-treat (population)
MOF: Multiple organ failure
MPI: Mannheim Peritonitis Index
NF: Nuclear factor
PCT: Procalcitonin
PP: Per protocol (population)
SOFA: Sequential Organ Failure Assessment
TNF: Tumor necrosis factor
