# Supplementary material for: Personalized medicine with IgGAM compared with standard of care for treatment of peritonitis after infectious source control (the PEPPER trial): study protocol for a randomized controlled trial
Source: Trials. 2019 Mar 4;20:156. doi: 10.1186/s13063-019-3244-4 (PMC6399861; doi:10.1186/s13063-019-3244-4)
Supplement: Supplementary file 1 — MOF score as modified by Lefering et al. [42]. (PDF 101 kb) [file 13063_2019_3244_MOESM1_ESM.pdf]

Langenbeck's Arch Surg (2002) 387:14–20
DOI 10.1007/s00423-001-0269-3

ORIGINAL ARTICLE

Rolf Lefering
R. Jan A. Goris
Ernst J. van Nieuwenhoven
Edmund Neugebauer

Revision of the multiple organ failure score

Received: 10 July 2001
Accepted: 19 November 2001
Published online: 31 January 2002
© Springer-Verlag 2002

Part of the results were presented at the 7th Vienna Shock Forum, Vienna/Austria, 1999

R. Lefering (✉) · E. Neugebauer
Biochemical and Experimental Division,
2nd Department of Surgery,
University of Cologne,
Ostmerheimer Strasse 200,
51109 Cologne, Germany
e-mail: r.lefering@uni-koeln.de
Tel.: +49-221-98957-16
Fax: +49-221-98957-30

R.J.A. Goris
Department of Surgery,
University Hospital Nijmegen St. Radboud,
Nijmegen, The Netherlands

E.J. van Nieuwenhoven
Afdeling Heelkunde, Canisius-Wilhelmina
Ziekenhuis, Nijmegen, The Netherlands

Abstract *Background and aim*: The multiple organ failure (MOF) score published by Goris et al. in 1985 was one of the first attempts to quantify severity of organ dysfunction and failure based on expert opinion in surgical intensive care unit patients. Fifteen years later a reassessment of this score is mandatory. *Patients and methods*: Daily MOF scores were documented in patients admitted to the surgical ICUs in Nijmegen (NL) and Cologne (D). Patients with an ICU stay ≤3 days were excluded. Organ dysfunction (1 point) and organ failure (2 points) were recorded for the following organ systems: lung, heart, kidney, liver, blood, gastrointestinal tract (GI), and central nervous system (CNS). Maximum scores were computed, and logistic regression analysis was used to optimize point weights for each organ system. Predictive power was analyzed using receiver operating characteristic (ROC) curves. *Results*: In all, 147 patients, mean age 56 years, were included with a total of 2,354 observation days. Hospital mortality was 30.6%. GI failure was present on only 3.3% of days, without impact on mortality. Valid evaluation of CNS was impossible in most cases due to sedation and ventilation. Reweighting of the score items revealed only marginal improvements in prediction. Mortality consistently increased with increase in number of failed organs. This phenomenon was even more pronounced in older patients, e.g., 55% mortality (age≥60) versus 0% (age<60) with two failing organs. *Conclusion*: Due to problems in definition and assessment (reliability) CNS and GI should not be considered in future assessments of the MOF score. The original point weights in the remaining five organ systems provide a valid and reliable risk stratification, at least in surgical ICU patients.

Keywords Intensive care · Multiple organ failure · Organ dysfunction · Score systems · Severity of illness index

Introduction

Organ dysfunction and failure is a leading cause of morbidity and mortality in intensive care. Monitoring organ function is thus part of everyday routine in the intensive care unit (ICU). This practice is also an important means for measuring outcome in research projects, clinical trials, and quality assessment programs. But a valid comparison of organ function among different patients, different hospitals, or even different disciplines requires the common use of a simple and reliable assessment of the patient's situation. Score systems that translate a complex clinical situation into a one dimensional point value have proven to be a helpful tool in these situations.

The multiple organ failure (MOF) score, published in 1985 by Goris et al. [1], was one of the first attempts to objectively quantify organ dysfunction in intensive care. Since then, it has been applied in a variety of clinical in-

Table 1 The multiple organ failure score. Definition of organ dsyfunction and failure according to Goris et al. [1]. *PEEP* positive end expiratory pressure, BP_{syst} systolic blood pressure, *SGOT* serum glutamic oxaloacetic transaminase

	Normal organ function<br>0 point	Organ dysfunction<br>1 point	Organ failure<br>2 points
Lung	No mechanical ventilation	Mechanical ventilation with PEEP≤10 and FiO_2≤0.4	Mechanical ventilation with PEEP >10 or FiO_2 >0.4
Heart	Normal blood pressure (BP_{syst})	BP_{syst}≥100 mmHg with low dose of vasoactive drugs[a]	Periods with BP_{syst} <100 mmHg and/or high dose of vasoactive drugs[b]
Kidney	Serum creatinine <2 mg/dl (<150 µmol/l)	Serum creatinine≥2 mg/dl (≥150 µmol/l)	Hemodialysis or peritoneal dialysis
Liver	Normal SGOT and bilirubin	SGOT≥25 units/l; bilirubin ≥2 mg/dl (≥ 34 µmol/l)	SGOT≥50 units/l; bilirubin ≥6 mg/dl (≥100 µmol/l)
Blood	Normal counts	Leukocytes ≥30,000; platelets ≤50,000	Leukocytes≥60,000 or ≤2,500
GI tract	Normal	Stress ulcer, Acalculous cholecystitis	Bleeding ulcer; Necrotizing enterocolitis and/or pancreatitis; perforation of gallbladder
CNS	Normal	Diminished responsiveness	Severely disturbed responsiveness; diffuse neuropathy

[a] Dopamine hydrochloride ≤10 µg/kg/min, or nitroglycerin of ≤20 µg/kg/min, or volume loading
[b] Dopamine hydrochloride >10 µg/kg/min, and/or nitroglycerin of >20 µg/kg/min

Table 2 Basic data of patients from both centers

	Total	Cologne	Nijmegen
Patients (*n*)	147	94	53
Age (mean, SD)	56 (19)	56 (17)	57 (21)
Sex (% male)	60.5%	61.7%	58.5%
Diagnosis (*n*, %)			
Gastrointestinal	64 (43%)	38 (40%)	26 (49%)
Trauma	39 (27%)	27 (29%)	12 (23%)
Vascular	23 (16%)	13 (14%)	10 (19%)
Other	21 (14%)	16 (17%)	5 (9%)
MOF score[a] on admission			
(Mean, SD)	3.1 (2.1)	2.6 (2.1)	4.1 (1.6)
ICU stay (mean, range)			
Survivor	18 (4–73)	18 (4–73)	18 (5–45)
Non-survivor	15 (4–34)	12 (4–32)	17 (5–34)
Total number of days	2348	1442	906
Hospital mortality (n, %)	45 (30.6%)	19 (20.2%)	26 (49.1%)

[a] Five organs, without gastrointestinal tract and central nervous system

vestigations [2, 3, 4, 5, 6, 7, 8]. Like one of the most recent attempts to quantify organ failure [9], however, the MOF score was based on the clinical judgment and the experience of experts only. A systematic evaluation of its validity for real patient data is necessary.

The present investigation was undertaken to answer the question of whether the original weights of 1 point for organ dysfunction and 2 points for organ failure (see Table 1) still reflect the importance of the different organs. Furthermore, the prospective data collection in two independent hospitals should give information about the reliability of the score and its components.

Materials and methods

Patients

During a 1-year period, 147 patients at risk for organ failure who needed intensive care for more than 3 days were included in a prospective observational cohort study in two centers. Fifty-three patients with at least one organ system failure were documented at the surgical ICU of the Academisch Ziekenhuis St. Radboud in Nijmegen, The Netherlands (May 1995 – April 1996). The intensive care unit of the 2nd Dept. of Surgery, University of Cologne, Germany, included 94 patients (Nov. 1995 – Oct. 1996). Both ICUs belong to tertiary-level surgical clinics in urban areas. Since data collection did not influence therapeutic decisions, informed consent was not deemed necessary. Data were collected and stored anonymously.

Most patients were admitted postoperatively. Basic characteristics of the 147 patients are presented in Table 2. The mortality in the subgroup from Nijmegen was higher since only patients who

Table 3 Modified MOF scores. Results of logistic regression analysis and derived score values for organ dysfunction (OD) and organ failure (OF) on day one ("early" MOF score, n=147), day 4 (n=147), and day 7 ("late" MOF score; n=115)

		Original score	Day 1		Day 4		Day 7	
			Coef.	Score	Coef.	Score	Coef.	Score
Lung	Dysfunction	1	0.5[a]	1	0.8	1	0.3[a]	0
	Failure	2	1.6	2	0.9	1	1.1	1
Liver	Dysfunction	1	0.7	1	0.9	1	0.9	1
	Failure	2	0.0[a]	1	0.6	1	1.5	2
Heart	Dysfunction	1	0.4[a]	1	0.6[a]	1	1.1	1
	Failure	2	1.4	2	1.7	2	1.4	2
Kidney	Dysfunction	1	1.9	2	1.6	2	1.6	2
	Failure	2	2.1	3	0.8[a]	2	1.0	2
Blood	Dysfunction	1	0.4[a]	1	0.7	1	0.6[a]	1
	Failure	2	2.4	3	6.4[a]	3	7.9[a]	3

[a] The value of the standard error exeeds the magnitude of the coefficient which indicates statistical uncertainty.

actually developed organ failure during ICU stay were included. Among the Cologne subgroup, six patients had no dysfunction or failure of the organs considered in the MOF score during ICU stay.

Data necessary for calculating the MOF score were collected daily from the patients' records for a total of 2,354 observation days by the responsible intensive care physicians. Organ dysfunction (1 point) and organ failure (2 points) were documented according to the published criteria for the following organ systems: lung, heart, kidney, liver, blood, gastrointestinal tract (GI), and central nervous system (CNS) [1] (Table 1). Documentation was continued until the patients left the ICU (except in four cases of patients who were transferred to another hospital and who required further intensive care). Patient status at hospital discharge was evaluated retrospectively, and outcome data (survival or death) were added to the database.

Comparative analysis of the prevalence of organ failure between the two hospitals revealed a systematic difference in two organ systems although patient characteristics were similar in both groups. First, a valid assessment of the CNS is difficult if the patient is intubated and sedated. While 91.3% of all days were scored zero in the Dutch patient group, this score was given on only 32.2% of days in the German group. On day 1, 70.2% of the German patients received points for CNS failure, in contrast to only 9.4% of the Dutch patients. Second, the definition of gastrointestinal dysfunction was interpreted in a broad way in the German subgroup (23.1% of all days) while kept strict in the Dutch subgroup (1.1% of all days). Furthermore, presence of GI failure had a limited influence on outcome in either subgroup of patients. As a consequence, the interdisciplinary study group decided to exclude these two organ systems from further analysis.

Statistics

Data were entered into a computer database and merged and analyzed with the statistical software package SPSS (vers. 9.9; SPSS Inc., Chicago, Ill., USA).

Maximum MOF score was defined as the highest daily MOF score observed during ICU stay. The aggregate maximum MOF score introduced by Moreno et al. [10] was defined as the sum of the worst values for each organ during ICU stay. Thus, the aggregate maximum MOF is at least as high as the usual maximum score, and it exceeds this value if organ failure occurs sequentially in one or more organ systems.

The non-parametric Mann-Whitney-U-Test was used for comparisons of the MOF score. Dichotomous data were compared with the Fisher exact test. A P-value of less than 0.05 was considered significant.

Logistic regression analysis with hospital mortality as dependent outcome parameter was applied to find new optimal point weights for the score items. Each organ system was included as an ordinal variable, so that separate point weights could be calculated for organ dysfunction and organ failure. In order to consider independent observations only, analysis was performed separately for all patients on day 1 (n=147), day 4 (n=147), and day 7 (n=115). The three analyses generated statistically optimal coefficients for organ dysfunction and organ failure, for each organ system. Subsequently, these coefficients were turned into integer values between 0 and 3 by a consensus process within the study group. For reasons of clinical consistency, organ failure was not allowed to receive fewer points than organ dysfunction. Thereby, three modified MOF scores (for day 1, day 4, and day 7) were generated, with the same definitions of organ dysfunction and organ failure, but with different point weights (Table 3).

The predictive power of the original as well as the modified scores was analyzed by calculating sensitivity and specificity for prediction of hospital outcome. Analysis was performed separately for score values from day 1 and day 7. Results were summarized by ROC curve analysis and the area under the curve (AUC) was calculated.

Results

Day of admission

On the day of ICU admission, mean MOF score was 3.1 points (range 0–9). Survivors had a lower MOF score on admission than non-survivors (2.5 vs. 4.6 points; P<0.001, U-Test). Organ failure as seen on day 1 is presented in Table 4, together with the associated mortality. Almost 60% of patients had organ failure for at least one organ on admission. The only patient with 9 points on admission was a 71-year-old woman with a fractured femur who had been resuscitated during operation. She died 5 days later. Eighteen patients had no signs of organ failure or organ dysfunction on admission; all of them survived. The Dutch subgroup showed higher MOF

Table 4 Prevalence of organ failure on the day of ICU admission (day 1), and occurence of organ failure during ICU stay. Hospital mortality and the day of first occurrence were calculated only for those patients who initially presented or developed that specific organ failure, respectively. For comparison, overall mortality rate in all 147 patients was 30.6%

	Organ failure on day 1		Organ failure during ICU stay		
	Patients	Mortality	Patients	Mortality	Day of first occurrence (mean, median)
Lung	52 (35%)	50%	94 (64%)	43%	2.8 (1)
Liver	36 (25%)	39%	87 (59%)	39%	5.1 (3)
Heart	47 (32%)	55%	81 (55%)	52%	2.2 (1)
Kidney	6 (4%)	67%	34 (23%)	71%	6.1 (4)
Blood	4 (3%)	75%	18 (12%)	78%	7.2 (5)
At least one organ	87 (59%)	46%	119 (81%)	38%	2.0 (1)

scores on admission than the German one (mean values 4.1 and 2.6, respectively; $P<0.001$, U-Test), which corresponds to a higher mortality rate in that group (49.1% vs. 20.2%, $P<0.001$, Fisher exact test).

Organ failure during ICU stay

The prevalence of organ dysfunction and organ failure among all observation days is presented in Fig. 1. The occurrence of organ failure during ICU stay as well as on admission was associated with a higher mortality rate as compared to the overall value of 30.6% As compared to the overall mortality rate (30.6%), mortality was higher if organ failure was either already present on admission or occurred during the stay in ICU. (Table 3). Twenty-eight patients had no organ failure during ICU stay, and seven of them also had no organ dysfunction (among the five organs considered in the MOF score). All these patients survived. Onset of organ failure varied among the organs considered. Among patients included as having developed heart or lung failure during ICU stay, over half of them were actually observed to be suffering from this condition on admission (median 1 day, Table 3), while the first occurrence of organ failure for the other three organs usually was observed later during ICU stay.

The maximum MOF score is strongly associated with mortality. None of the five patients with a five-organ failure survived (maximum MOF score = 10) while only one out of 56 patients with a maximum MOF score of 3 points or below died (Fig. 2). Maximum MOF score was reached on average 3.9 days after admission in survivors and 6.7 days after admission in non-survivors. The remaining ICU stay after the maximum MOF score had been reached averaged 14.0 and 8.3 days for survivors and nonsurvivors, respectively. The aggregate maximum MOF score was 0.56 points higher on average than the observed maximum MOF score (mean values 5.22 and 4.66, respectively). Higher values for the aggregate score was noted in 41.5% of patients, identical values in the rest. Distribution of the two maximum scores together with their associated mortality rates are given in Fig. 2.

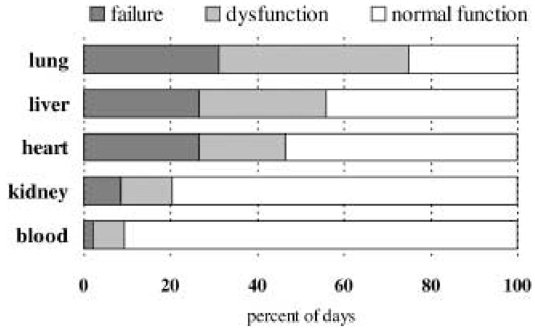

Fig. 1 Prevalence of days with organ failure and organ dysfuction in patients with more than 3 days of ICU care, based on all 2,348 observation days

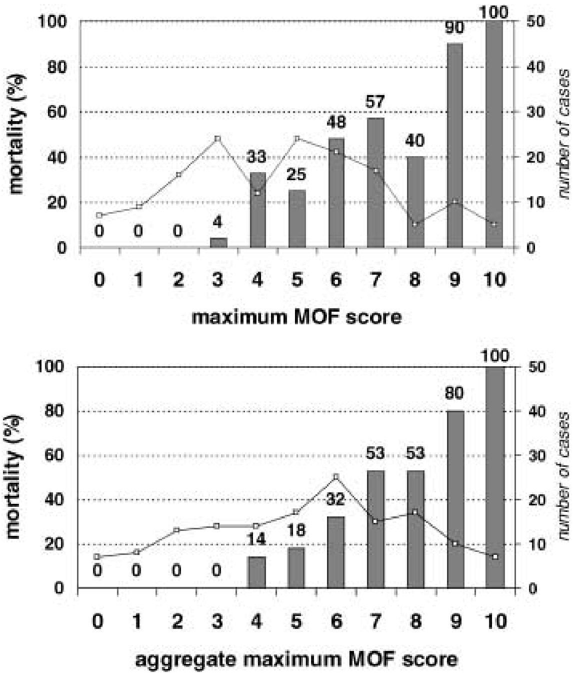

Fig. 2 Maximum multiple organ failure (*MOF*) and aggregate maximum MOF score (sum of worst values for each organ system during ICU stay) and associated mortality in 147 patients. The number of patients in each category is indicated by the *line*

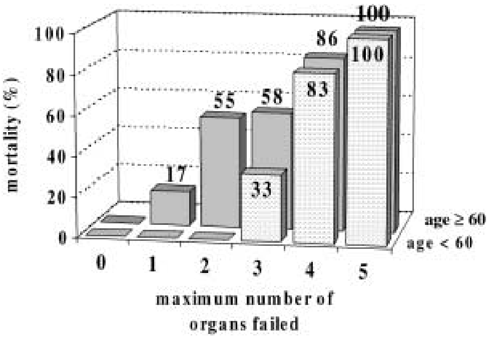

Fig. 3 Mortality according to maximum number of organs failed during ICU stay for two age groups

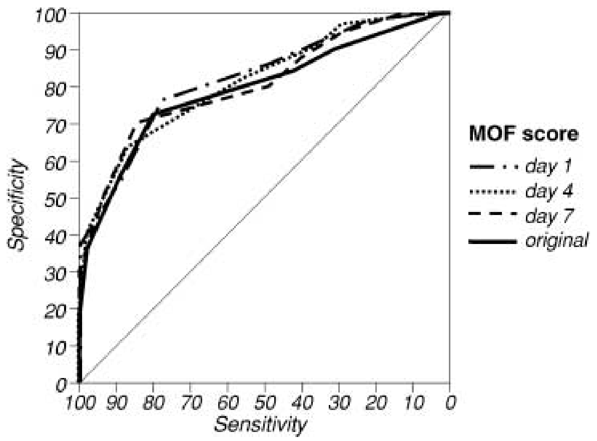

Fig. 4 ROC curve analysis based on data from the first day (n=147) for the original as well as for three modified multiple organ failure (*MOF*) scores (day 1. day 4, and day 7; for definition see Table 4). The areas under the curve were 0.794 for the original MOF score, and 0.825, 0.813, and 0.811 for the three modified scores, respectively.

The same consistent increase in mortality was observed with the number of organs failed: mortality rates were 0%, 11%; 30%; 48%; 85%, and 100% for none to five organs, respectively. Patient age was also observed to be an important influence on mortality. Patients under 60 years of age had a significantly lower mortality rate (18.5%; n=65) than elderly patients (40.2%; n=82, P=0.007, Fisher exact test) although their initial MOF scores were nearly comparable (2.9 versus 3.3 points; P=0.18). The maximum MOF scores attained during ICU stay were also similar (4.5 and 4.8 points, respectively; P=0.41). Main differences were observed in patients with two or three organ failures (Fig. 3).

Modification of point values

Multivariate analysis of score values based on data from day 1, day 4, and day 7 revealed a decreasing importance of the status of the lung over time, fewer points for liver failure, and an increase in points for kidney dysfunction and failure (Table 4). Comparison of predictive power with ROC curve analysis revealed that the modified "early" MOF score (see Table 4, column "day 1") was superior to the original score using day 1 data (AUC 0.825 versus 0.794, respectively; Fig. 4), and that the modified "late" MOF score (see Table 4, column "day 7") performed better on day 7 data (AUC 0.807 versus 0.792), as expected. However, the differences observed were small in comparison to the 95% confidence interval for the original score on day 1 [0.722–0.867] and day 7 [0.703–0.882].

Discussion

The monitoring of organ failure with scoring systems has become an established procedure in clinical trials and comparative analyses in intensive care [11]. Along with the definition of organ failure given by Knaus et al. [12], the MOF score of Goris et al. [1] was one of the first attempts to create an objective point system. Several MOF scores followed [9, 13] using a variety of definitions [14]. However, any score needs a regular evaluation of its validity and reliability. Now, 15 years after the first publication of the MOF score, a re-evaluation was deemed necessary, and to be based on data from real intensive care patients.

A first important observation was made due to data collection being performed independently in two separate environments. Although basic characteristics of patients and the manuals for scoring were identical, interobserver variability was found to be substantial in two organ systems: the CNS and the GI tract. Regarding the CNS, a valid assessment of the mental function is usually not possible in sedated and ventilated ICU patients. This is a well-known problem of intensive care severity scores [15, 16]. The Glasgow Coma Scale (GCS), initially designed to describe the prognosis of patients with severe head injuries, confirmed its usefulness in this patient group [17]. However, its integration into scoring systems for monitoring intensive care in general seems to introduce more problems than benefits. Although guidelines for scoring exist, different strategies are followed in reality [18]. Also as part of other ICU scoring systems, the GCS was identified as the major source of interobserver variation [15]. In consequence, the study group decided not only to exclude the CNS from the present analyses but also to suggest performing future assessments without regarding the CNS. This does, of course, not mean that unconsciousness is irrelevant for describing a patient's situation, but the aim of a score system is to provide an objective and reproducible tool that can be applied in different settings with high reliability. Therefore, assessment of CNS should not be included in daily MOF score unless a simple and robust parameter for measuring mental function in ICU patients becomes available.

A similar problem has been observed with GI failure, because it lacked a clear definition. Furthermore, the number of patients with GI failure, using its original definition, was low and its occurrence was rarely associated with outcome. We support the arguments of Marshall et al. [13], who, after a thorough evaluation of the literature, decided not to include GI failure in their multiple organ dysfunction score (MODS). None of the parameters suggested for quantifying GI failure fulfilled the methodological criteria given in that paper.

The primary aim of our study was to evaluate the score points given for organ dysfunction and organ failure. In this sense, patients without any signs of organ failure are not helpful and were thus not documented in the Dutch center. The requirement of at least 4 days in ICU should help to select the relevant patient group. A few patients from the German subgroup did not receive any MOF points during their ICU stay, but we decided to include them in the analysis to have at least some information about low score values. Of course, in this area data are statistically weak and should be interpreted very carefully.

Our study population is a selected group of patients who were more severely ill than an average ICU population, and the reported prevalence rates of organ failure can not directly be generalized. Very severely ill patients who died within a few days as well as surviving patients who required intensive care for only a short period of time have been excluded. However, neither of these groups of patients is the target population for continuous daily monitoring of organ failure. This aspect also has to be considered when measures of predictive ability such as the area under the ROC curve are compared to published values of other authors.

The attempt to improve the score by attributing new point weights to the different levels of organ function was not very successful. Although some improvements seemed to be observed, the overall effect was small. Of course, a statistically optimized formula can easily achieve results superior to a predefined score, as can be seen by the improvements in the areas under the ROC curves. These improvements are marginal, they are statistically uncertain, and they are likely to disappear when applied to a new independent population, however. It is well known that a predictive model performs best on the data for which it was developed and may lose predictive power when applied to new circumstances – or cases. Therefore, it was the decision of the study group to keep the original equal weights for dysfunction (1 point) and failure (2 points) for all organs.

The attempt to define “early” and “late” MOF scores highlights another interesting fact of sequential organ failure monitoring. Lung failure and heart failure, if present, were often observed very early after admission to ICU. In contrast, onset of liver failure was usually seen later. Moreno et al. also observed that maximum scores for liver were not reached until day 5, on average [10]. Interestingly, in the logistic organ dysfunction (LOD) system [19] liver failure can add only one point at most to the total score (range 0–22). This is due to the fact the LOD was based on thousands of data collected on the first day of ICU stay only. Thus, LOD would seem to be more an initial classification tool for severity of disease such as Simplified Acute Physiology Score SAPS [20] than a score for monitoring organ function.

Outcome evaluation with a MOF score, e.g., in clinical trials, requires a reduction of sequential daily values into a summary measure for each patient. The maximum score attained during ICU stay is such a summary measure, the aggregate maximum score (sum of the worst values for each organ) is another one. In our data the aggregate maximum MOF score showed a slightly more consistent increase in mortality rate with increasing point values (Fig. 2).

Finally, the important influence of age on prognosis is very impressively demonstrated in the present investigation when using the organ failure definition of the MOF score with only five organs. While organ failure of four or five organs has a very bad prognosis, irrespective of age, mortality is remarkably increased in patients over 60 if only two or three organs fail. This again highlights the fact that score values alone do not reflect the situation as a whole, but can only give a reduced view of reality.

Conclusion

Monitoring of CNS and GI tract failure leads to a loss of reliability and have been discarded in the revised MOF score. The points for dysfunction (1 point) and failure (2 points) for the remaining five organs seem to be reasonable since possible improvements were considered to be of questionable value. Daily assessment of the MOF score allows for a valid and objective monitoring of organ dysfunction and organ failure, and the aggregate maximum MOF score is a valid summary measure for outcome evaluation.

References

1. Goris RJA, te Boekhorst TPA, Nuytinck JKS, Gimbrère JSF (1985) Multiple-organ failure. Generalized autodestructive inflammation? Arch Surg 120: 1109–1115
2. Pacher R, Redl H, Frass M, Petzl DH, Schuster E, Woloszczuk W (1989) Relationship between neopterin and granulocyte elastase plasma levels and the severity of multiple organ failure. Crit Care Med 17: 221–226
3. Roumen RM, Schers TJ, de Boer HH, Goris RJ (1992) Scoring systems for predicting outcome in acute hemorrhagic necrotizing pancreatitis. Eur J Surg 158: 167–171

4. Roumen RM, Hendriks T, van der Ven-Jongekrijg J, Nieuwenhuijzen GA Sauerwein RW, van der Meer JW, Goris RJ (1993) Cytokine patterns in patients after major vascular surgery, hemorrhagic shock, and severe blunt trauma. Relation with subsequent adult respiratory distress syndrome and multiple organ failure. Ann Surg 218: 769–776
5. Pilz G, Kaab S, Kreuzer E, Werdan K (1994) Evaluation of definitions and parameters for sepsis assessment in patients after cardiac surgery. Infection 22: 8–17
6. Dominioni L, Chiappa A, Bianchi V, Interdonato PF, Festi L, Carcano G, Benevento A, Dionigi R (1997) Infected pancreatic necrosis complicated by multiple organ failure. Hepatogastroenterology 44: 968–974
7. Bastian L, Weimann A, Bischoff W, Meier PN, Grotz M, Stan C, Regel G (1998) Klinische Auswirkungen einer supplementierten enteralen Nährlösung beim schweren Polytrauma. Unfallchirurg 101: 105–114
8. Ishikawa K, Tanaka H, Matsuoka T, Shimazu T, Yoshioka T, Sugimoto H (1998) Recombinant human granulocyte colony-stimulating factor attenuates inflammatory responses in septic patients with neutropenia. J Trauma 44: 1047–1054
9. Vincent JL, Moreno R, Takala J, Willatts S, De Mendoca A, Bruining H, Reinhart CK, Suter PM, Thijs LG (1996) The SOFA (sepsis-related organ failure assessment) score to describe organ dysfunction/failure. Intensive Care Med 22: 707–710
10. Moreno R, Vincent JL, Matos R, Mendonca A, Cantraine F, Thijs L, Takala J, Sprung C, Antonelli M, Bruining H, Willatts S (1999) The use of maximum SOFA score to quantify organ dysfunction/failure in intensive care. Results of a prospective, multicentre study. Working Group on Sepsis related Problems of the ESICM. Intensive Care Med 25: 686–696
11. Marshall JC (1999) Organ dysfunction as an outcome measure in clinical trials. Europ J Surg 165 (Suppl. 584): 62–67
12. Knaus WA, Draper EA, Wagner DP, Zimmerman JE (1985) Prognosis of acute organ-system failure. Ann Surg 202: 685–693
13. Marshall JC, Cook DJ, Christou NV, Bernard GR, Sprung CL, Sibbald WJ (1995) Multiple organ dysfunction score: a reliable descriptor of a complex clinical outcome. Crit Care Med 23: 1638–1652
14. Bertleff MJOE, Bruining HA (1997) How should multiple organ dysfunction syndrome be assessed? A review of the variations in current scoring systems. Eur J Surg 163: 405–409
15. Chen LM, Martin CM, Morrison TL, Sibbald WJ (1999) Interobserver variability in data collection of the APACHE II score in teaching and community hospitals. Crit Care Med 27: 1999–2004
16. Livingston BM, Mackenzie SJ, MacKirdy FN, Howie JC (2000) Should the pre-sedation Glasgow Coma Scale value be used when calculating Acute Physiology and Chronic Health Evaluation scores for sedated patients? Crit Care Med 28: 389–394
17. Cho DY, Wang YC (1997) Comparison of the Apache III, Apache II and Glasgow Coma Scale in acute head injury for prediction of mortality and functional outcome. Intensive Care Med 23: 77–84
18. Marion DW, Carlier PM (1994) Problems with initial Glasgow Coma Scale assessment caused by prehospital treatment of patients with head injuries: results of a national survey. J Trauma 36: 89–95
19. Le Gall JR, Klar J, Lemeshow S, Saulnier F, Alberti C, Artigas A, Teres D (1996) The logistic organ dysfunction system. A new way to assess organ dysfunction in the intensive care unit. JAMA 276: 802–810
20. Le Gall JR, Lemeshow S, Saulnier F (1993) A new simplified acute physiology score (SAPS II) based on a European / North American multicenter study. JAMA 270: 2957–2963
